# Four new species of *Fissocantharis* Pic, 1921 (Coleoptera, Cantharidae) from China

**DOI:** 10.3897/zookeys.738.19884

**Published:** 2018-02-19

**Authors:** Yuxia Yang, Yaqing Qi, Xingke Yang

**Affiliations:** 1 The Key Laboratory of Zoological Systematics and Application, College of Life Sciences, Hebei University, Baoding 071002, Hebei Province, China; 2 Key Laboratory of Zoological Systematics and Evolution, Institute of Zoology, Chinese Academy of Sciences, Beijing 100101, China

**Keywords:** East Asia, description, Cantharinae, taxonomy

## Abstract

Four new species of *Fissocantharis* Pic, 1921 are described: *F. securiclata* **sp. n.** (China: Zhejiang, Anhui), *F. maculicollis* **sp. n.** (China: Zhejiang), *F. hainana* **sp. n.** (China: Hainan) and *F. laticollis* **sp. n.** (China: Hainan), which are illustrated with the habitus and aedeagus of the male, the abdominal sternite VIII and the internal genitalia of the female. A key to the species from southeast China is provided.

## Introduction

The taxonomy of the Chinese species of *Fissocantharis* Pic, 1921 (sensu Yang et al. 2009) was studied mainly by Wittmer in the last half century. Among his publications, the one, in which Wittmer (1988) revised the species from mainland China and adjacent areas, is especially useful for our study of species from southeast China. We recently added some species from Fujian, Zhejiang, Hunan, Anhui, Guangxi, Guangdong and Hainan (Yang and Yang 2009, Yang et al. 2014, 2015a, b). In the present study, four new species were discovered and described here under the names *F. securiclata* sp. n. (China: Zhejiang, Anhui), *F. maculicollis*sp. n. (China: Zhejiang), *F. hainana* sp. n. (China: Hainan) and *F. laticollis* sp. n. (China: Hainan).

## Material and methods

The material is preserved in the following collections: Institute of Zoology, Chinese Academy of Sciences, Beijing, China (IZAS); Museum of Hebei University, Baoding, China (MHBU); and the private collection of Mr. Andreas Pütz, Germany (CoAP).

Genitalia of both sexes and abdominal sternites VIII of females were dissected and cleared in 10% KOH solution, and female genitalia were dyed with hematoxylin. Habitus photos were taken by a Leica M205A stereomicroscope, multiple layers were stacked using Combine ZM (Helicon Focus 5.3). Line drawings were made using a camera lucida attached to a Nikon SMZ1500 stereomicroscope, then edited in CorelDRAW 12 and Adobe Photoshop 8.0.1.

**Figure 1.**
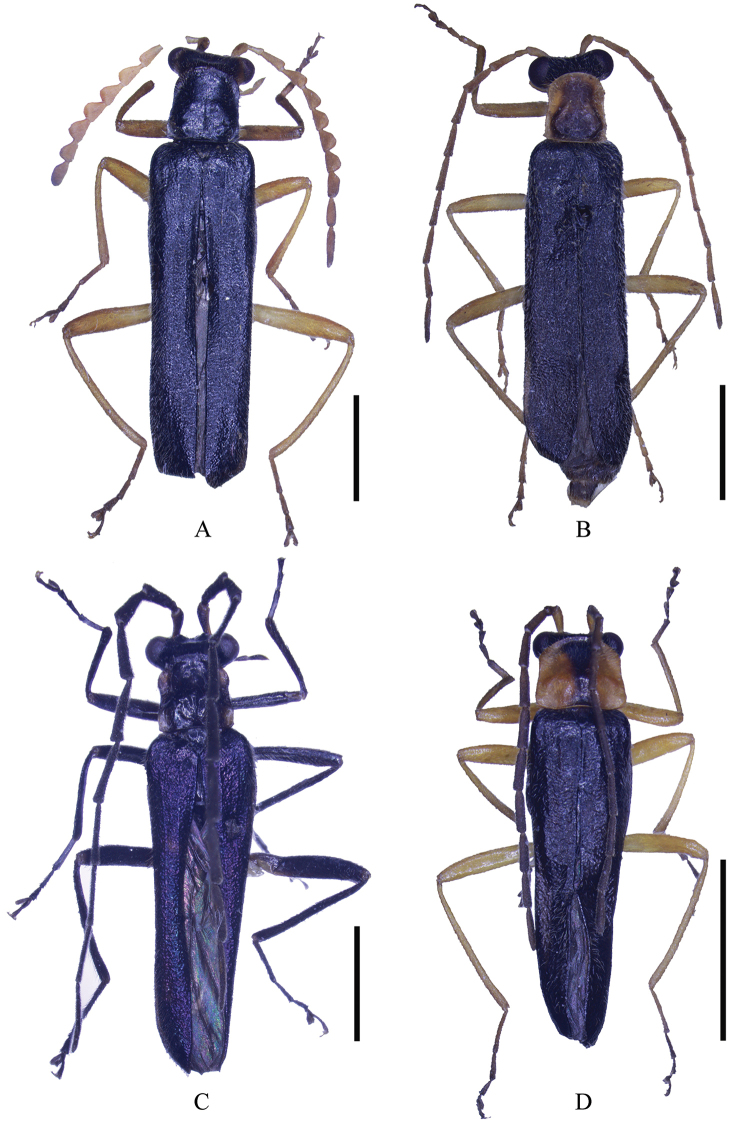
Male habitus (holotype), dorsal view: **A** *Fissocantharis securiclata* sp. n. **B** *F. maculicollis* sp. n. **C** *F. hainana* sp. n. **D** *F. laticollis* sp. n. Scale bars: 2.0 mm.

Complete label data in Chinese are transliterated for type specimens, and quotation marks are used for labels in English. Body length was measured from the anterior edge of the clypeus to the elytral apex and body width across the humeri of elytra. Morphological terminology of female genitalia follows Brancucci (1980). The specimens are prepared with the antennae extended posterad and photographed in dorsal view (Fig. 1). The lateral edge is the narrow surface of the antenna seen in this position from lateral view.

## Taxonomy

### Key to the species of *Fissocantharis* (males) from southeast China

**Table:** 

1	Elytra red-brown or yellow-brown	**2**
–	Elytra black or dark purple or blue	**3**
2	Antennomeres III–VI with longitudinal ridges along lateral edges, VI–VIII each with a deep and oblong fovea (Wittmer 1997: fig. 178); pronotum slightly longer than wide, with posterior angles obtusely rectangular	***F. pieli* (Pic, 1937)**
–	Antennomeres IV–XI with longitudinal impressions along lateral edges (Yang et al. 2014: fig. 10); pronotum wider than long, with posterior angles triangular and sharp	***F. acuticollis* Y. Yang & X. Yang, 2014**
3	Antennae filiform or subfiliform with middle antennomeres nearly parallel-sided or at most obliquely widened apically	**4**
–	Middle antennomeres strongly deformed in various ways, unlike above	**16**
4	Antennae simple	**5**
–	Middle antennomeres with impressions or ridges or bulges along lateral edges	**9**
5	Pronotum wider than long, about 1.2 times as wide as long	***F. laticollis* sp. n.**
–	Pronotum longer than wide or subequal, at most 1.1 times as long as wide	**6**
6	Pronotum uniformly black	**7**
–	Pronotum yellow, with a large black marking on disc	***F. maculicollis* sp. n.**
7	Head uniformly yellow	**8**
–	Head mostly black	***F. paulioincrassata* (Wittmer, 1951)**
8	Maxillary palpomeres II–IV dorsoventrally flattened and widened, II convex at basal part of dorsal side	***F. latipalpa* Y. Yang & X. Yang, 2015**
–	Maxillary palpomeres II–IV slender, not flattened or widened	***F. pallidiceps* (Pic, 1911)**
9	Middle antennomeres with longitudinal costa-like ridges along lateral edges	**10**
–	Middle antennomeres with smooth impressions or scar-like bulges along lateral edges	**11**
10	Antennae slightly cylindrically thickened, antennomeres III–IX with longitudinal ridges along lateral edges; aedeagus: conjoint dorsal plate of parameres longer than ventral process of each paramere	***F. buonloiensis* Wittmer, 1993**
–	Antennae slightly dorsoventrally flattened, antennomeres III–VIII with longitudinal ridges along lateral edges; aedeagus: conjoint dorsal plate of parameres shorter than ventral process of each paramere	***F. sexcostata* Y. Yang & X. Yang, 2015**
11	Middle antennomeres with oblong scar-like bulges along lateral edges	**12**
–	Middle antennomeres with longitudinal or oblong or round impressions along lateral edges	**13**
12	Aedeagus: conjoint dorsal plate of parameres with apical edge rounded in middle, ventral processes abruptly narrowed apically	***F. gracilipes* (Pic, 1927)**
–	Aedeagus: conjoint dorsal plate of parameres with apical edge acute in middle, ventral processes evenly narrowed apically	***F. eschara* Y. Yang & X. Yang, 2015**
13	Antennomeres III–X parallel-sided	***F. sinensomima* Y. Yang & X. Yang, 2015**
–	Antennomeres III–X slightly dorsoventrally flattened and obliquely widened apically	**14**
14	Aedeagus: conjoint dorsal plate of parameres greatly reduced, with apical edge nearly horizontally aligned with bases of ventral process of each paramere	***F. basilaris* Y. Yang & X. Yang, 2015**
–	Aedeagus: conjoint dorsal plate of parameres moderately developed, with apical edge distinctly protuberant over bases of ventral process of each paramere	**15**
15	Aedeagus: conjoint dorsal plate of parameres about half length of ventral process of each paramere, nearly parallel-sided at basal part	***F. sinensis* (Wittmer, 1988)**
–	Aedeagus: conjoint dorsal plate of parameres about one-third length of ventral process of each paramere, tapered apically	***F. hainana* sp. n.**
16	Antennomeres III–IV or to V deformed, cylindrically thickened or excavate	**17**
–	Antennomeres III–VIII or to IX, X, XI deformed, at least one antennomere not parallel-sided, or excavate or emarginate	**20**
17	Maxillary palpomeres II–III excavate	**18**
–	Maxillary palpi not excavate	**19**
18	Antennomere IV with two projections at basal part (Yang et al. 2015b: fig. 7)	***F. biprojicientis* Y. Yang & X. Yang, 2015**
–	Antennomere IV unlike above, without projections (Wittmer 1988: fig. 4)	***F. bidifformis* (Wittmer, 1988)**
19	Antennomere III excavate along the whole length of lateral edge, IV distinctly wider than III (Wittmer 1988: fig. 1)	***F. similis* (Wittmer, 1951)**
–	Antennomere III excavate at apex, IV as wide as III (Wittmer 1988: fig. 2)	***F. tridifformis* (Wittmer, 1988)**
20	Antennomere XI knife-like, with lateral edge triangularly protuberant near basal one-fourth part, obliquely narrowed apically, acute at apex (Wittmer 1988: fig. 12)	**21**
–	Antennomere XI parallel-sided	**23**
21	Antennomeres XI about one-third longer than X	***F. angusta* (Fairmaire, 1900)**
–	Antennomeres XI about as twice long as X	**22**
22	Antennae and legs mostly black; aedeagus: conjoint dorsal plates of parameres with apical edge straight	***F. tachulanensis* (Wittmer, 1988)**
–	Antennae and legs mostly orange; aedeagus: conjoint dorsal plates of parameres with apical edge rounded	***F. flavimembris* (Wittmer, 1951)**
23	Antennomere IX distinctly lengthened and widened, about twice as long as wide	**24**
–	Antennomere IX unlike above	**25**
24	Antennomere IX with lateral edges triangularly protuberant and slightly curled dorsally in middle (Yang et al. 2015b: fig. 2A)	***F. novemexcavata* (Wittmer, 1951)**
–	Antennomere IX parallel-sided (Yang et al. 2015b: fig. 2F)	***F. novemoblonga* Y. Yang & X. Yang, 2015**
25	Antennomere IV distinctly cylindrically thickened and excavate along the whole length of lateral edge, width wider ventrally than dorsally, V–X with apical angles truncate and finely bordered (Yang and Yang 2009: figs 3–6)	***F. imparicornis* (Y. Yang & X. Yang, 2009)**
–	Antennomeres IV–X unlike above	**26**
26	Antennomeres III–VIII each emarginate at apical part of lateral edge (Wittmer 1988: fig. 16)	***F. multiexcavata* (Wittmer, 1988)**
–	Antennomeres III–VIII unlike above	**27**
27	Antennomeres VIII or and VII projecting laterally	**28**
–	Antennomeres VII and VIII unlike above	**29**
28	Antennomere VIII with apical angle strongly projecting laterally, III–VIII minutely serrated along lateral edges (Wittmer 1988: fig. 13)	***F. flavicornis* (Gorham, 1889)**
–	Antennomeres VII–VIII with apical angles moderately projecting laterally, III–VIII not serrated along lateral edges (Wittmer 1988: fig. 14)	***F. cicatricosa* (Wittmer, 1988)**
29	Antennomeres III–X obliquely widened apically (Wittmer 1989: fig. 8); aedeagus: conjoint dorsal plate of parameres greatly reduced, with apical edge nearly horizontally aligned with bases of ventral processes	***F. liuchowensis* (Wittmer, 1989)**
–	Antennomere III obliquely widened apically, IV–IX securiform, which abruptly widened apically and rounded at apical angles (fig. 1); aedeagus: conjoint dorsal plate of parameres moderately developed, with apical edge distinctly protuberant over bases of ventral processes	***F. securiclata* sp. n.**

**Remarks.** In the key, *F. bothridera* (Fairmaire, 1887) (located in Fujian) is not included, because it was described only on the female form, which is difficult to distinguish among other female *Fissocantharis* (Wittmer 1988). A checklist of the species from southeast China is provided after the descriptions, except for those from Guangxi, which have been listed by Yang et al. (2015b).

### Descriptions of new species

#### Fissocantharis securiclata

Taxon classificationAnimaliaColeopteraCantharidae

Y. Yang & X. Yang sp. n.

http://zoobank.org/84FD8C2E-9B44-488C-9EBA-2CFF4205E868

**Figure 2.**
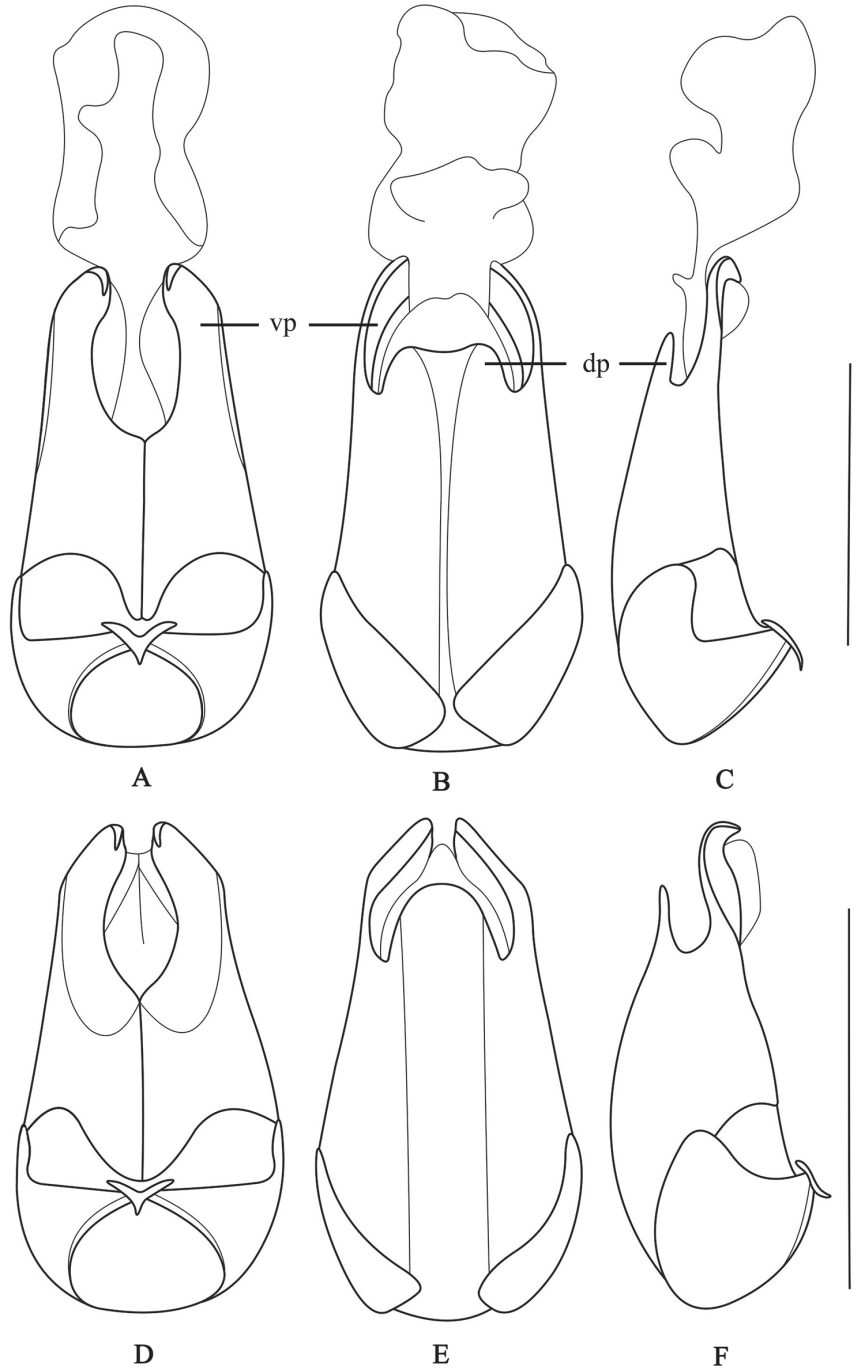
Aedeagus (**A, D** ventral view **B, E** dorsal view **C, F** lateral view): **A–C** *Fissocantharis securiclata* sp. n. **D–F** *F. maculicollis* sp. n. Scale bars: 1.0 mm. (dp: conjoint dorsal plate of parameres; vp: ventral process of each paramere).

Figs 1A , 2A–C , 4A , 5A

##### Type material.

Holotype ♂ (MHBU): CHINA, Anhui, Jixi, Qingliangfeng, 2–5.VI.2013, leg. J.S. Xu & C.X. Yuan. Paratypes: CHINA: 1♀ (MHBU): same data as holotype; Zhejiang: 1♀ (IZAS): “Tienmu Shan, 10.6.1936, O. Piel coll.”; 1♂ (IZAS): “Tienmu Shan, 6.6.1936, O. Piel coll.”; 1♂ (CoAP) “CHINA: Zhejiang [CH07-39], Hangzhou Pref., Tianmu Shan, 40 km WNW Linan, water reservoir, 30°20'56"N, 119°18'42"E, 300 m, plant refuse, litter from rock edges, 17.VI.2007, leg. A. Pütz”.

##### Distribution.

China (Anhui, Zhejiang).

##### Description.

***Male*** (Fig. 1A). Head orange, darkened at both sides of vertex, mouthparts orange, darkened at mandibular apices, terminal maxillary and labial palpomeres and antennae orange, darkened dorsally at antennomeres III–IX, pronotum, scutellum and elytra black, legs orange, darkened at tarsi, ventral surface of body black, yellow at posterior and sides of abdominal sternites. Body densely covered with short recumbent gray pubescence.

Head subquadrate, narrowed posteriorly behind eyes; eyes moderately projecting, head width across eyes greater than that of anterior edge of pronotum; apical maxillary palpomeres nearly long-triangular, widest at apical two-fifths, acute at apices; antennae extending to elytral midlength, antennomeres II about twice as long as wide, III obliquely widened apically, truncate at apical angles, about twice as long as wide, IV–IX securiform, abruptly widened apically and rounded at apical angles, IV and IX slightly longer than wide, V–VIII nearly as long as wide, X and XI nearly parallel-sided, XI pointed at apices and about 1.5 times as long as X.

Pronotum subquadrate, about 1.1 times longer than wide, anterior edge arcuate, sides slightly diverging posteriorly, posterior edge nearly straight, anterior angles rounded, posterior angles nearly rectangular, disc slightly convex at posterolateral parts, surface finely and densely punctate.

Elytra about 3.0 times longer than wide, 4.0 times longer than pronotum, width at humeri greater than posterior edge of pronotum, sides nearly parallel, surface slightly more coarsely and densely punctate than pronotum.

Legs with all tarsal claws bifid, each with lower projection as long as upper one.

Aedeagus (Fig. 2A–C): ventral process of each paramere wide, slightly narrowed apically and hooked at apex; conjoint dorsal plate of parameres moderately developed, about one-third length of ventral process of each paramere, slightly narrowed apically, with apex medially concave.

***Female.*** Similar to male, but vertex black, antennae black, antenomeres I and II orange, filiform and simple, extending to basal third of elytra. Abdominal sternite VIII (Fig. 4A) nearly straight at posterior edge, lateral angles obtuse.

Internal genitalia (Fig. 5A): vagina abruptly extended apically as a short and thick duct at ventroapical portion; diverticulum and spermathecal duct arising from end of duct of vagina; diverticulum evenly thin, spiraled and long; spermathecal duct slightly shorter and thicker than diverticulum; spermatheca nearly as long as and slightly thicker than diverticulum, with a moderately long thin accessory gland, which is longer than spermatheca.

Body length: 7.9–9.0 mm; width: 1.7–2.0 mm.

##### Etymology.

The specific name is derived from Latin *securiclatus* (axe-like), referring to its antennomeres IV–IX securiform in male, which abruptly widened apically and rounded at apical angles.

##### Diagnosis.

Head mostly orange, pronotum and elytra uniformly black; male antennomeres IV–IX securiform, abruptly widened apically and rounded at apical angles; aedeagus: conjoint dorsal plate of parameres moderately developed, about one-third length of ventral process of each paramere, slightly narrowed apically, with apical edge medially concave.

##### Remarks.

No other species has the male antennomeres IV–IX securiform as in this species. This species is similar to *F. pallidiceps* (Pic, 1911) in the body coloration, but can be easily distinguished by the male antennae, of which the antennomeres III–IX are widened apically, while simple filiform in *F. pallidiceps* (Wittmer 1988: fig. 6); aedeagus: the conjoint dorsal plate of parameres is slightly narrowed apically, with the apex medially concave, while distinctly narrowed apically, with the apex straight in *F. pallidiceps* (Wittmer 1988: fig. 26). It also resembles *F. liuchowensis* (Wittmer, 1989), but can be differentiated by the male antennae, of which the antennomeres IV–IX are abruptly widened apically and rounded at apical angles, while obliquely widened apically in *F. liuchowensis* (Wittmer 1989: fig. 8); aedeagus: the conjoint dorsal plate of parameres is moderately developed, while greatly reduced in *F. liuchowensis* (Wittmer 1989: fig. 9).

#### Fissocantharis maculicollis

Taxon classificationAnimaliaColeopteraCantharidae

Y. Yang & X. Yang sp. n.

http://zoobank.org/CA4F3AAE-29F9-4AC8-A554-F7853A89AA09

Figs 1B , 2D–F , 4B , 5B

##### Type material.

Holotype ♂ (MHBU): CHINA, Zhejiang, Qingliangfeng, Longtangshan, 19.V.2011, leg. G.L. Xie. Paratypes: CHINA, Zhejiang: 2♂♂, 1♀ (IZAS): Xitianmushan, Xianrending, 1500m, 6.VI.1998, leg. H. Wu; 1♂ (IZAS): Xitianmushan, Kaishanlaodian, 1050m, 30.V.1998, leg. M.S. Zhao; 1♂ (IZAS): Xitianmushan, Dahenglu, 1200m, 6.VI.1998, leg. M.S. Zhao; 1♂ (IZAS): Qingyuan, Baishanzu, 20.VIII.1993, leg. H. Wu.

##### Distribution.

China (Zhejiang).

##### Description.

***Male*** (Fig. 1B). Head yellow, vertex black, mouthparts yellow, darkened at mandibular apices, antennae black, antennomeres I and II yellow, prothorax yellow, pronotum with a large dark brown marking on disc, marking extending to posterior but anterior edge or sides, and its posterior part wider than anterior part, scutellum and elytra black, legs yellow, slightly darkened at tarsi, ventral surface of body black. Body densely covered with short recumbent gray pubescence.

Head subquadrate, narrowed posteriorly behind eyes; eyes strongly projecting, head width across eyes greater than that of anterior edge of pronotum; apical maxillary palpomeres nearly long-triangular, widest at apical two-fifths, acute at apices; antennae filiform and simple, extending to apical third of elytra, antennomeres II about 1.5 times as long as wide, III about 3.0 times as long as II, IV–X subequal in length, XI pointed at apices and slightly longer than X.

Pronotum subquadrate, about 1.1 times longer than wide, anterior edge rounded, sides slightly diverging posteriorly and sinuate, posterior edge nearly straight, anterior angles rounded, posterior angles nearly rectangular, disc convex at posterolateral parts, surface finely and densely punctate.

Elytra about 3.5 times as long as wide, 5.0 times as long as pronotum, width at humeri greater than posterior edge of pronotum, sides nearly parallel, surface slightly more coarsely and densely punctate than pronotum.

Legs with all tarsal claws bifid, each with lower projection as long as upper one.

Aedeagus (Fig. 2D–F): ventral process of each paramere wide, slightly narrowed apically and hooked at apex; conjoint dorsal plate of parameres moderately developed, about a half length of ventral process of each paramere, with apical edge rounded.

***Female.*** Similar to male, but eyes only slightly projecting, antennae narrower and shorter, extending to basal third of elytra, pronotum slightly convex at posterolateral parts. Abdominal sternite VIII (Fig. 4B) widely and shallowly emarginate at middle of posterior edge, bottom of middle emargination slightly roundly protuberant in middle, lateral angles slightly acute.

Internal genitalia (Fig. 5B): vagina abruptly extended apically as a short and thick duct at ventroapical portion; diverticulum and spermathecal duct arising from end of duct of vagina; diverticulum thick at basal portion and thinned apically, spiraled and moderately long; spermathecal duct nearly as long as and thinner than basal portion of diverticulum; spermatheca much longer than and nearly as thick as apical portion of diverticulum, with a moderately long thin accessory gland, which slightly shorter than spermatheca.

##### Variation within type series.

Male antennae mostly yellow in some throughout antennomere or ventrally. Body length: 6.2–7.4 mm; width: 1.2–1.6 mm.

##### Etymology.

The specific name is derived from the Latin *macula* (marking) and *collum* (neck), referring to its pronotum with a black marking on disc.

##### Diagnosis.

Elytra black, prothorax yellow, pronotum with a large dark brown marking on disc; male antennae filiform and simple; aedeagus: conjoint dorsal plate of parameres moderately developed, about a half length of ventral process of each paramere, with apical edge rounded.

##### Remarks.

This new species is similar to *F. nigriceps* Y. Yang & Okushima, 2016 (located in Taiwan) in the body shape and male antennae, but can be distinguished from the latter by the following characters: the pronotum has a black marking on the disc and the aedeagus has a wider conjoint dorsal plate of parameres, while in *F. nigriceps*, the pronotum is uniformly orange and the aedeagus has a narrower conjoint dorsal plate of parameres (Li et al. 2016: figs 6G–I, 12A). It is also resembles *F. paulioincrassata* (Wittmer, 1951) in the male antennae, but differs in having a yellow pronotum, with a large black marking on the disc, while uniformly black in *F. paulioincrassata*; aedeagus: the conjoint dorsal plate of parameres is moderately developed, about half length of ventral process of each paramere, while slightly shorter, about one-third length in *F. paulioincrassata* (Wittmer 1988: 353).

#### Fissocantharis hainana

Taxon classificationAnimaliaColeopteraCantharidae

Y. Yang & X. Yang sp. n.

http://zoobank.org/471AF09B-DEA7-437C-8CCE-D9D1928DE8A1

**Figure 3.**
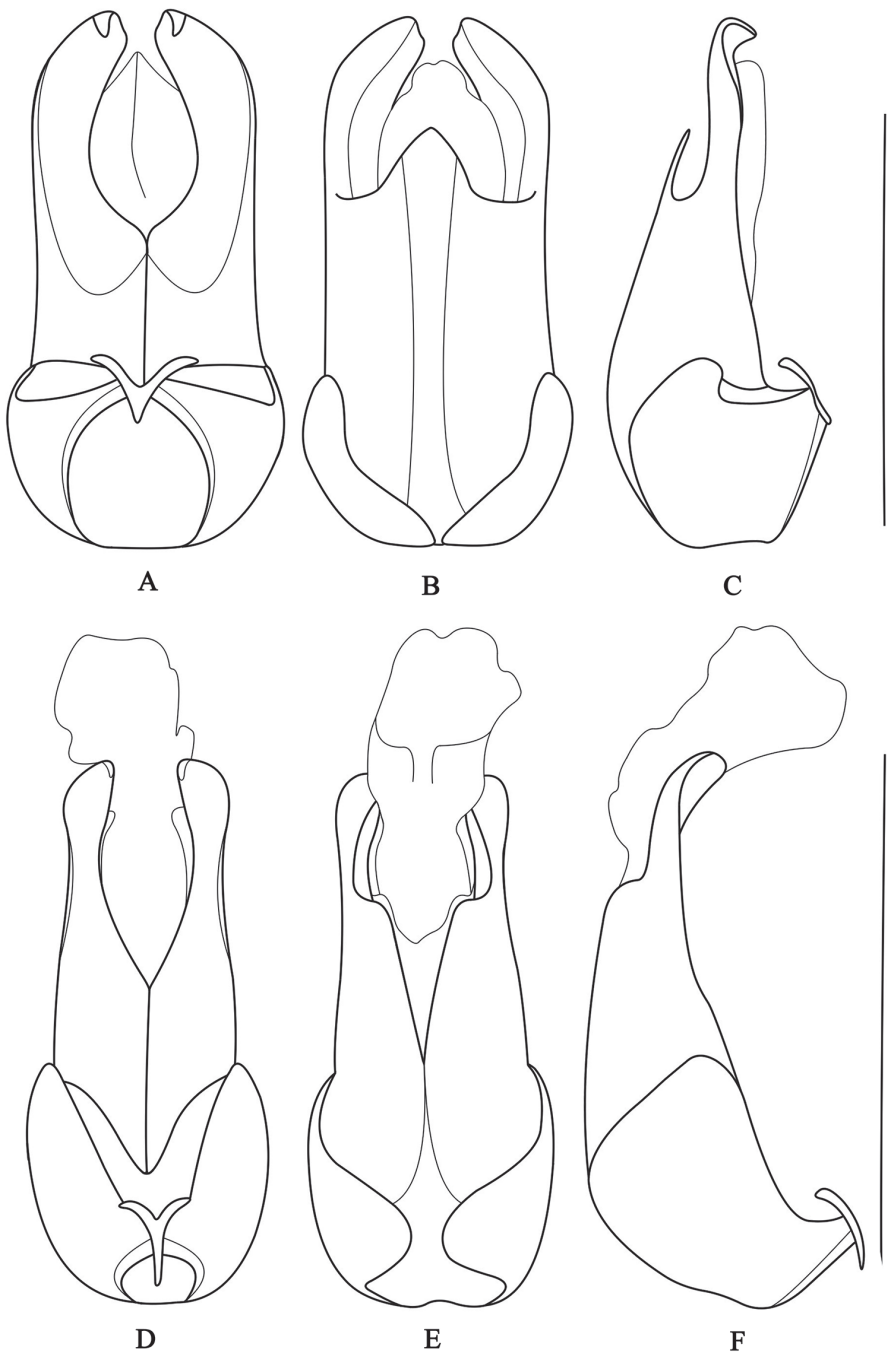
Aedeagus (**A, D** ventral view **B, E** dorsal view **C, F** lateral view): **A–C** *Fissocantharis hainana* sp. n. **D–F** *F. laticollis* sp. n. Scale bars: 1.0 mm.

**Figure 4.**
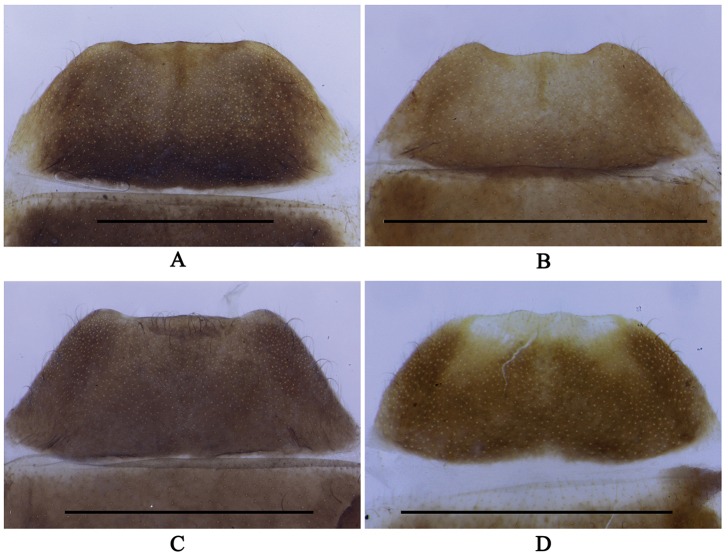
Abdominal sternite VIII of female, ventral view: **A** *Fissocantharis securiclata* sp. n. **B** *F. maculicollis* sp. n. **C** *F. hainana* sp. n. **D** *F. laticollis* sp. n. Scale bars: 1.0 mm.

**Figure 5.**
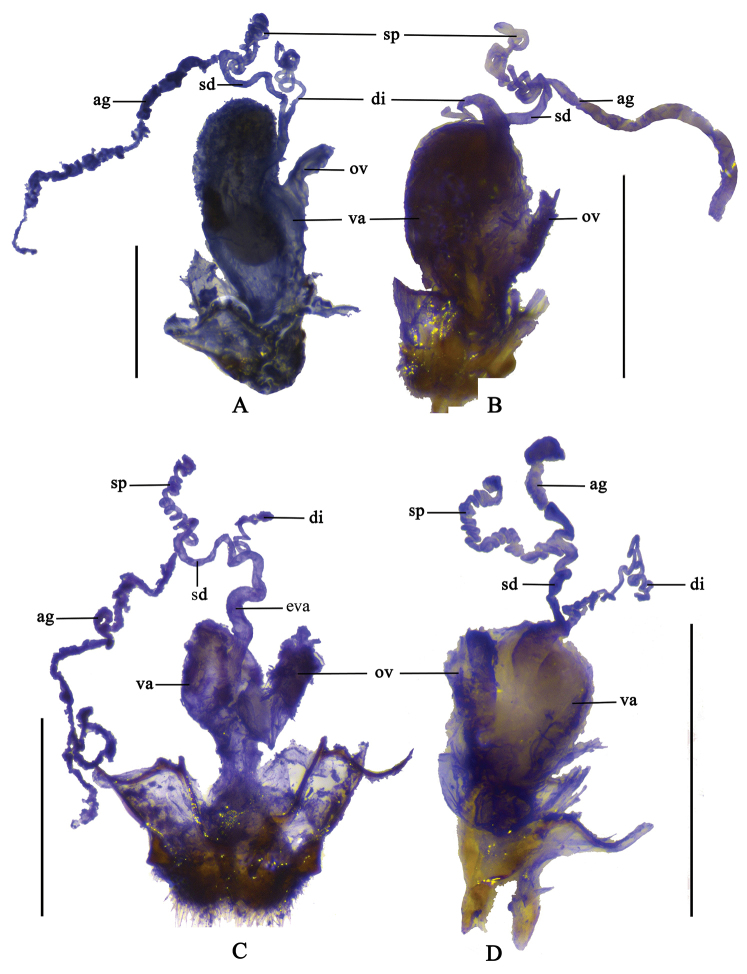
Female internal genitalia, lateral view: **A** *Fissocantharis securiclata* sp. n. **B** *F. maculicollis* sp. n. **C** *F. hainana* sp. n. **D** *F. laticollis* sp. n. Scale bars: 1.0 mm. (ag: accessory gland; di: diverticulum; eva: extension of vagina; sd: spermathecal duct; sp: spermatheca; ov: median oviduct; va: vagina).

Figs 1C , 3A–C , 4C , 5C

##### Type material.

Holotype ♂ (IZAS): CHINA, Hainan, Diaoluoshan, 928m, 30.III.1980, leg. Y.X. Yang. Paratypes: CHINA, Hainan: 1♂, 1♀ (IZAS): same data as holotype; 1♀ (IZAS): same locality, 31.III.2008, leg. L.Y. Jiang; 1♀ (IZAS): Qiongzhong, Wuzhishan, 800m, date and collector unknown; 1♀ (IZAS): Qiongzhong, 800m, 5.IV.1980, collector unknown; 1♂ (IZAS): Wuzhishan, 936.8m, 18.54°N, 109.40°E, 2.IV.2008, leg. X. L. Chen; 1♀ (IZAS): Wuzhishan, 708–1206m, 18.89°N, 109.69°E, 9.IV.2010, leg. K.Y. Zhang; 1♂, 1♀ (IZAS): Qiongzhong, Limushan, 840m, 19.17°N, 109.72°E, 6.IV.2010, K.Y. Zhang; 1♂, 2♀♀ (IZAS): Baisha, Yinggeling, Nankai, 336m, 19.00°N, 109.22°E, 6.IV.2010, leg. M.Y. Lin; 3♀♀ (IZAS): “Hainan Prov., Lingshui, Diaoluoshan, Xin-an, 18.72510°N, 109.86861°E, 921m, 2007.03.25, Shi H.L., Yuan F. coll.”.

##### Distribution.

China (Hainan).

##### Description.

***Male*** (Fig. 1C). Body black, except mandibles brown, darkened at apices, prothorax orange, pronotum with a large black marking on disc, marking extending to anterior and posterior edges except at sides, and its posterior part wider than anterior part, elytra dark purple, with metallic reflection.

Head subquadrate, narrowed posteriorly behind eyes; eyes strongly projecting, head width across eyes wider than that of anterior edge of pronotum; apical maxillary palpomeres nearly long-triangular, widest at apical two-fifths, acute at apices; antennae nearly extending to elytral apices, antennomeres II nearly as long as wide, III–IX dorsoventrally flattened and obliquely widened apically, III about 3.0 times as long as II, IV–X subequal in length, X and XI parallel-sided, XI pointed at apices and slightly longer than X, III–XI each with a round or oblong smooth impression near middle of lateral edge.

Pronotum subquadrate, nearly as long as wide, widest near base, anterior edge rounded, sides sinuate, posterior edge nearly straight, anterior angles rounded, posterior angles nearly rectangular, disc convex at posterolateral parts, surface finely and densely punctate.

Elytra about 3.0 times longer than wide, 4.0 times longer than pronotum, width at humeri greater than posterior edge of pronotum, sides nearly parallel, surface slightly more coarsely and densely punctate than pronotum.

Legs with all tarsal claws bifid, each with lower projection as long as upper one.

Aedeagus (Fig. 3A–C): ventral process of each paramere wide, slightly narrowed apically and hooked at apex; conjoint dorsal plate of parameres moderately developed, about one-third length of ventral process of each paramere, with apical edge acute in middle.

***Female.*** Similar to male, but eyes only slightly projecting, antennae filiform and simple, extending to midlength of elytra, pronotum moderately convex at posterolateral parts. Abdominal sternite VIII (Fig. 4C) widely and shallowly emarginate at middle of posterior edge, bottom of middle emargination nearly straight, lateral angles rounded.

Internal genitalia (Fig. 5C): vagina abruptly extended apically as a long and thick duct at ventroapical portion; diverticulum and spermathecal duct arising from end of long duct of vagina; diverticulum thin, spiraled and moderately long; spermathecal duct nearly as long as and slightly thicker than diverticulum; spermatheca longer and slightly thicker than diverticulum, with a very long and thin accessory gland, which much longer than spermatheca.

##### Variation within type series.

Pronotum orange in some, only darkened at anterior and posterior margins, elytra dark blue, or with weakly metallic reflection. Body length: 7.0–11.5 mm; width: 1.3– 2.2 mm.

##### Etymology.

The specific name is derived from the type locality, Hainan Province, China.

##### Diagnosis.

Elytra dark purple, with metallic reflection; male antennae subfiliform, antennomeres III–IX dorsoventrally flattened and obliquely widened apically, III–XI each with a round or oblong smooth impression near middle of lateral edge; aedeagus: conjoint dorsal plate of parameres moderately developed, about one-third length of ventral process of each paramere, with apical edge acute in middle.

##### Remarks.

The new species is similar to *F. langana* (Pic, 1923) (located in N. Vietnam), *F. langaniformis* (Wittmer, 1989) (N. Vietnam) and *F. sinensis* (Wittmer, 1988) in the body coloration and male antennae, but can be distinguished by the structure of the aedeagus: the conjoint dorsal plate of parameres is moderately developed, about one-third length of ventral process of each paramere, with the apical edge acute in the middle, while it is greatly reduced in *F. langana* (Wittmer 1988: fig. 29) and *F. langaniformis* (Wittmer 1989: fig. 10), and about half length of ventral process in *F. sinensis* (Yang et al. 2009: fig. 3A–C).

#### Fissocantharis laticollis

Taxon classificationAnimaliaColeopteraCantharidae

Y. Yang & X. Yang sp. n.

http://zoobank.org/573DA6BD-6CE7-4861-B8E5-29D720ABA13D

Figs 1D , 3D–F , 4D , 5D

##### Type material.

Holotype ♂ (IZAS): CHINA, Hainan, Jianfengling, Tianchi, 24.III.2008, leg. Y.X. Yang. Paratypes: CHINA, Hainan: 6♂♂, 3♀♀ (IZAS): same data as holotype; 5♂♂, 1♀ (IZAS): same locality and collector, 812m, 23.III.2008; 4♂♂, 10♀♀ (IZAS): Wenchang, Tongguling, 317m, 18.III.2008, leg. Y.X. Yang; 1♂, 4♀♀ (IZAS): Wuzhishan, 708–1206 m, 18.89°N, 109.69°E, leg. M.Y. Lin; 1♀ (IZAS): Jianfeng, Tianchi, 750m, 19.III.1980, leg. S.Y. Wang; 1♂ (IZAS): Jianfeng, 760m, 20.III.1980, collector unknown; 1♂ (IZAS): Qiongzhong, Wuzhishan, 800m, 6.IV.1980, leg. S.Y. Wang; 2♂♂ (IZAS): Qiongzhong, 1840m, 5.IV.1980, collector unknown.

##### Distribution.

China (Hainan).

##### Description.

***Male*** (Fig. 1D). Head orange, vertex black, mouthparts orange, darkened at mandibular apices, terminal maxillary and labial palpomeres, antennae black, antennomeres I and II orange at ventral sides, prothorax orange, pronotum with an inverted triangular black marking at middle of anterior part on disc, scutellum and elytra black, legs orange, darkened at tarsi, ventral surface of body black, yellow at posterior edges and sides of abdominal ventrites. Body densely covered with short recumbent gray pubescence.

Head rounded, slightly narrowed posteriorly behind eyes; eyes moderately projecting, head width across eyes slightly wider than that of anterior edge of pronotum; apical maxillary palpomeres nearly long-triangular, widest at apical two-fifths length, acute at apices; antennae filiform and slightly thickened, extending to apical third of elytra, antenomeres II slightly longer than wide, III about twice as long as II, IV–X subequal in length, XI pointed at apices and slightly longer than X.

Pronotum transverse, about 1.2 times wider than long, anterior edge rounded, sides sinuate, slightly converging posteriorly at anterior one-third length, then slightly diverging posteriorly at posterior part, posterior edge slightly arcuate, anterior angles subrounded, posterior angles nearly rectangular, disc slightly convex at posterolateral parts, surface finely and densely punctate.

Elytra about 3.5 times as long as wide, 4.5 times as long as pronotum, width at humeri slightly greater than posterior edge of pronotum, sides nearly parallel, surface slightly more coarsely and densely punctate than on pronotum.

Legs with all tarsal claws bifid, each with lower projection as long as upper one.

Aedeagus (Fig. 3D–F): ventral process of each paramere wide, narrowed apically and hardly hooked at apex; conjoint dorsal plate of parameres reduced, with apical edge deeply emarginate in middle.

***Female.*** Similar to male, but eyes slightly projecting, head width across eyes nearly as wide as anterior edge of pronotum, antennae narrower and shorter, extending to midlength of elytra. Abdominal sternite VIII (Fig. 4D) with a pair of small bilobate protuberances in middle of posterior edge, lateral angles obtuse.

Internal genitalia (Fig. 5D): vagina abruptly extended apically as a short and thick duct; diverticulum and spermathecal duct arising from end of duct of vagina; diverticulum evenly thin, long and spiral; spermathecal duct shorter and thicker than diverticulum; spermatheca nearly as long as and slightly thicker than diverticulum, with a short thin accessory gland, which much shorter than spermatheca.

Body length: 4.0–5.5 mm; width: 0.8–1.1 mm.

##### Etymology.

The specific name is derived from the Latin *latus* (wide) and *collum* (neck), referring to its pronotum wider than long.

##### Diagnosis.

Elytra black; male antennae filiform and simple; pronotum transverse, about 1.2 times wider than long, sides sinuate, slightly converging posteriorly at anterior one-third length, then slightly diverging posteriorly at posterior part; aedeagus: conjoint dorsal plate of parameres reduced, with apical edge deeply emarginate in middle.

##### Remarks.

This species could be distinguished by its pronotum, which is distinctly wider than long, while always longer than wide or subequal in length in other species of *Fissocantharis*. It is more similar to *F. imparicornis* (Y. Yang & X. Yang, 2009) (China: Hainan) in body coloration, but differs from the latter by the following characters: the male antennae are simple and the aedeagus has a reduced conjoint dorsal plate of parameres, while in *F. imparicornis*, the middle antennomeres of the male are deformed and the aedeagus has a well-developed conjoint dorsal plate of parameres.(Yang and Yang 2009: figs 3–6, 8).

### Other species from southeast China (except those from Guangxi)

***Fissocantharis acuticollis* Y. Yang & X. Yang, 2014**

*Fissocantharis acuticollis* Y. Yang & X. Yang, 2014, Yang et al. 2014: 53, figs 2, 5, 10, 20–22.

**Distribution.** China (Zhejiang, Fujian, Guangdong, Hunan).

***Fissocantharis bothridera* (Fairmaire, 1887)**

*Rhagonycha bothridera* Fairmaire, 1887: 125, 127.

*Rhagonycha nigricoloriceps* Pic, 1928: 55. Synonymized by Wittmer 1988: 344.

*Micropodabrus bothriderus*: Wittmer 1988: 344.

*Fissocantharis bothridera*: Yang et al. 2009: 49.

**Distribution.** China (Fujian).

***Fissocantharis flavimembris* (Wittmer, 1951)**

*Podabrus flavimembris* Wittmer, 1951: 96, fig. 1.

*Micropodabrus flavimembris*: Wittmer 1988: 358.

*Fissocantharis flavimembris*: Yang et al. 2009: 49.

**Distribution.** China (Fujian).

***Fissocantharis imparicornis* (Y. Yang & X. Yang, 2009)**

*Micropodabrus imparicornis* Y. Yang & X. Yang, 2009: 65, figs 1–9.

*Fissocantharis imparicornis*: Yang et al. 2009: 49.

**Distribution.** China (Hainan).

***Fissocantharis novemexcavata* (Wittmer, 1951)**

*Podabrus novemexcavatus* Wittmer, 1951: 97, fig. 3.

Podabrus novemexcavatus ab. testaceicollis Wittmer, 1951: 97, fig. 3. Synonymized by Kazantsev and Brancucci 2007: 257.

*Micropodabrus novemexcavatus*: Wittmer 1988: 359.

*Fissocantharis novemexcavata*: Yang et al. 2009: 49; Yang et al. 2015b: 363, figs 2A, 3A–C, 5A, 6A.

**Distribution.** China (Fujian).

***Fissocantharis novemoblonga* Y. Yang & X. Yang, 2015**

*Fissocantharis novemoblonga* Y. Yang & X. Yang, 2015, Yang et al. 2015b: 371, figs 1D, 2F, 4G–I, 5C, 6C.

**Distribution.** China (Zhejiang, Anhui).

***Fissocantharis pauloincrassata* (Wittmer, 1951)**

*Podabrus pauloincrassatus* Wittmer, 1951: 97.

*Micropodabrus pauloincrassatus*: Wittmer 1988: 353.

*Fissocantharis pauloincrassata*: Yang et al. 2009: 49.

**Distribution.** China (Fujian).

***Fissocantharis pallidiceps* (Pic, 1911)**

*Rhagonycha pallidiceps* Pic, 1911: 271.

*Micropodabrus pallidiceps*: Wittmer 1988: 353.

*Fissocantharis pallidiceps*: Yang et al. 2009: 49.

**Distribution.** China (Zhejiang).

***Fissocantharis pieli* (Pic, 1937)**

*Lycocerus pieli* Pic, 1937: 172.

*Micropodabrus pieli*: Wittmer 1997: 312, figs 178–180.

*Fissocantharis pieli*: Yang et al. 2009: 49; Yang et al. 2014: 47, figs 3, 6, 11–13.

**Distribution.** China (Zhejiang, Fujian).

***Fissocantharis similis* (Wittmer, 1951)**

*Podabrus similis* Wittmer, 1951: 97.

*Micropodabrus similis*: Wittmer 1988: 348.

*Fissocantharis similis*: Yang et al. 2009: 49.

**Distribution.** China (Fujian).

## Supplementary Material

XML Treatment for Fissocantharis securiclata

XML Treatment for Fissocantharis maculicollis

XML Treatment for Fissocantharis hainana

XML Treatment for Fissocantharis laticollis
